# Unequal high streets? A spatial analysis of inequalities in health-related amenities in England from 2014-2024

**DOI:** 10.1016/j.socscimed.2025.118863

**Published:** 2025-12-04

**Authors:** Eman Zied Abozied, Luke Munford, Adam Todd, Clare Bambra

**Affiliations:** a Population Health Science Institute, Newcastle University, Newcastle upon Tyne, NE1 7RU United Kingdom; b Division of Population Health, Health Services Research and Primary Care, University of Manchester, Oxford Road, Manchester, M13 9PL, United Kingdom; c School of Pharmacy, Newcastle University, Newcastle upon Tyne, NE1 7RU, United Kingdom

## Abstract

There are persistent inequalities in health-related behaviours in England which are stratified by region and deprivation. These are influenced by the interaction of people with places they live in, over and above individual risk factors. The amenities available on high streets is one such aspect of place but to date, its role in shaping health behaviours has been under examined.

Our study presents a novel analysis into health-related amenities and change in availability between 2014 and 2024. We used geographic data analysis and statistical modelling of Ordnance Survey Points of Interest data to describe the association between health-related amenities, area level deprivation and region across England.

We found that there were significant inequalities in amenity availability by deprivation over the past decade. The most deprived areas were more likely to gain a takeaway (OR1.56[1.28,1.91]), a bookmaker (OR2.14[1.64,2.79]), or a vape shop (OR2.11[1.68, 2.66] and more likely to lose a supermarket (OR1.95[1.62,2.35]) or a public toilet (OR1.34[1.12,1.62]), compared to the least deprived areas. These patterns were similar on a regional level - the North was more likely to gain a takeaway (OR1.65[1.44,1.90]), a bookmaker (OR1.40[1.18,1.66]), a pawnbroker (OR1.72[1.23,2.40]) or a vape shop (OR1.30[1.11,1.51]) compared to the South.

Our findings indicate that the most deprived areas and regions are gaining potentially health-harming amenities and losing health-conducive ones. Through this analysis, we argue that amenities that facilitate health behaviours on high streets are influenced by political and economic drivers of health inequalities and therefore require political decisions to manage, rather than individual behaviour change.

## 1 Introduction

Health outcomes are influenced by the interaction of people with the places they live in, over and above individual risk factors (Macintyre and Ellaway, 2003). Places are primarily constructed through the composition (demographics) of their residents, the context (built environment, infrastructure) and vertical drivers (macro social, economic and political decisions) (Bambra et al., 2019; Cummins et al., 2007). There are persistent health inequalities in England which are stratified by region and deprivation. Health is generally worse in the Northern regions and in deprived and “left behind” areas (Bambra et al., 2014, p. 2; Pike et al., 2023). The primary drivers of such health inequalities are structural factors - the social, economic and political decisions leading to the uneven distribution of wealth and resources, rather than individual behaviours or characteristics (Bambra et al., 2019). Such structural factors can also affect the retail environment of places, and subsequently the amenities available on high streets.

The availability of health-related amenities on the high street (the services and shops available) can provide opportunities for health promoting or health reducing behaviours. Specific amenities, such as takeaways, gambling outlets, and alcohol only outlets, represent ‘environmental risk factors’ for unhealthy behaviours and subsequent poor health outcomes (Macdonald et al., 2018; Shortt et al., 2015; Wardle et al., 2014). On the other hand, amenities such as supermarkets, pharmacies, and public toilets can influence health for the better (Lamichhane et al., 2013; Maguire et al., 2015). The decision to undertake specific health behaviours is often automatic rather than conscious and linked to the socio-economic conditions of a place and the availability of amenities (Marek et al., 2021; Ziauddeen et al., 2018).

Previous research has studied one or two types of amenities, and usually in one time point or location (for examples, see: Burgoine et al., 2016; Macdonald et al., 2018; Shortt et al., 2015). There has been less focus on the availability of multiple types of outlets, the change in availability over time and the association with socio-economic deprivation and geographical region in England. To our knowledge, no studies have explored trends over time in changes to the availability of health-related amenities across all of England or studied the distribution of amenities by geographic region. Our study presents a novel analysis into health-related amenities on the high street and their change in availability over the past decade. We use the most up to date yearly data from the Ordnance Survey Points of Interest database which has seldom been used in health research. Through linking amenity availability to deprivation, region and change over time, we argue that health-related amenities on the high streets are driven by similar social, political and economic vertical drivers of health inequalities and not only by individual demand of such amenities.

We start by reviewing the existing literature on how the built environment can influence health, the upstream drivers of environmental and health inequalities, high streets and infrastructure inequalities and the influence of specific amenities on health across space and time. Then, we detail the methods employed in the study, namely geographic and statistical analysis. We then present our results which indicated that there are significant inequalities in amenity availability, with the most deprived areas more likely to gain some types of unhealthy amenities, and more likely to lose healthy ones, when compared to the least deprived areas. Additionally, we present how the spatial patterning of health-related amenities is similar to that of existing geographic health inequalities. Finally, we discuss how our findings relate to the literature, offer some reflections on the limitations of the research, and consider policy implications for our findings.

## 2 Background

The characteristics of a place have long been known to influence health, over and above individual factors (Jones and Moon, 1993; Macintyre and Ellaway, 2003). The healthfulness of an area is determined by the composition (the people who live there: their demographics, socioeconomic status and health behaviours) and the context of a place (the nature of the place itself: built and natural environment, availability of amenities, political, social and economic infrastructures) (Macintyre et al., 2002). More important than context or composition alone is the relational aspect of places, which are the sum of interactions between people and place, and how places shape people and vice versa. This concept is not just in the health literature outlined by Cummins et al. (2007) but also in urban planning where it is referred to as the space-society paradigm or the social production of space (Hillier et al., 1993; Lefebvre, 1991).

Places are also constructed through vertical drivers (macro conditions) of wider political, environmental and economic decisions. Such drivers include decisions such as national funding strategies or tax rates, and unforeseen circumstances such as recessions and pandemics. These vertical drivers influence the context and composition of a place, and are largely outside the control of individuals and local authorities (Bambra et al., 2019). Further, places are influenced through subtle means of social capital and power dynamics, whereby some populations are able to effect changes to their places, which has knock on effects on the distribution of infrastructure and other resources (Harvey, 1996).

Health can be influenced by place through direct exposure (for example, air pollution) or indirectly through the availability of resources and opportunities for residents. Places can provide ‘opportunity structures’ (Macintyre et al., 2002), a ‘positive affordance’ (Mittelmark et al., 2022, p. 266) or therapeutic (health promoting) value (Bell et al., 2018) to people living in these places, which influences their health outcomes. The availability of amenities (and the health behaviours this enables or constrains) is an indirect effect of place on health.

There are significant, persisting health inequalities across England, with health being worse in the more deprived areas and coastal areas as well as regionally within the North-South health divide (Bambra et al., 2014). Such geographic health inequalities are primarily determined by structural and political factors such as the uneven distribution of wealth and resources, rather than individual behaviours or characteristics. Often, this uneven distribution is a deliberate political and economic choice, operating at a national or transnational scale (Bambra et al., 2019). Such health inequalities are prevalent in “left behind areas” which also suffer from economic and social deprivation (Pike et al., 2023). Places are left behind due to political choices, such as austerity and welfare reform (Beatty and Fothergill, 2018), and “spatially differentiated economic change” including deindustrialisation and lack of subsequent opportunities (Martin et al., 2021, p. 55). This has resulted in economic turmoil, wealth inequalities, increased socio-economic deprivation and subsequent negative effects on health and wellbeing. Being ‘left behind’ can also result in spatial stigma, which is the over-association of places with negative descriptors such as high levels of criminality, risk and danger. This stigma contributes to further inequalities (Keene and Padilla, 2014) and poorer health outcomes (Link and Phelan, 2001), on top of those associated with socio-economic deprivation.

Similar structural factors also determine the allocation of infrastructure, which is often not equitably distributed (Latham and Layton, 2019). High streets (and the amenities present on them) are considered community infrastructure and have existed since the inception of human settlement. They are primarily based on connectivity of the street network and are considered to be the spatial and economic heart of settlements (Griffiths, 2015). In recent years however, high streets have become more financialised, with a focus on retail over the community aspects (Bayliss et al., 2023), leading to further socio-spatial inequalities in the city.

Part of the fragility of high streets is related to financialisation which makes high streets more vulnerable to vertical macro-economic and political change. The uneven distribution of resources creates differentiation in the retail environment based on vertical forces rather than the demands and needs of residents. An example of this is the decline of independent, community based businesses and the increase in transnational retail chains on high streets, the distribution of which is driven by global market forces. Due to this uneven change and lack of meeting resident’s needs, in several places in the UK, town centres and high streets have become stigmatised places and associated with decline and lack of safety (Garthwaite and Bambra, 2018; Pattison, 2023). Changes in retail behaviours (to online shopping – exacerbated by the pandemic lock downs) and declining incomes in more deprived areas (as a result of wage stagnation, austerity and higher economic inactivity since 2008) has also added further to the decline of the English high street.

Increased socio-economic deprivation is associated with several health-harming outcomes including lower diet quality (Santa-Ramírez et al., 2025), increased smoking prevalence (Duncan et al., 1999) and problem gambling behaviour (Rintoul et al., 2013). Regions which are left behind, such as the North of England, also had higher rates of smoking and heavy drinking (Beard et al., 2017). As well as being associated with health-harming outcomes, socio-economic deprivation of an area can also drive the types of amenities present there, whether through purposeful targeting in the case of gambling outlets, pawn-brokers and fast food (Adeniyi et al., 2023; Cummins et al., 2005) or the reduction of other types of retail due to economic viability. ‘Environmental bads’ such as gambling outlets, alcohol and tobacco retailers, are also more common in more deprived areas (Saunders et al., 2023; Shortt et al., 2015), leading to higher rates of health harming behaviours (Connor et al., 2011; Pearce et al., 2016; Shortt et al., 2016). Deprived areas are often also obesogenic environments, with high availability of takeaway outlets and lower availability of supermarkets, leading to higher rates of obesity (Burgoine et al., 2016; Keeble et al., 2021; Maguire et al., 2017). This effect is not limited to physical health – the availability of pawnbrokers and gambling outlets has also been linked to poorer mental health outcomes (Eisenberg-Guyot et al., 2018; Pearce et al., 2008). The lack of health conducive amenities such as supermarkets and public toilets also vary by deprivation and constrain health behaviours such as access to healthy food and hygiene (Kelly, 2024; Mahony et al., 2025). This represents a form of ‘environmental injustice’, in which availability of health-related amenities compounds the health effects on already deprived populations (Brulle and Pellow, 2006; Murray et al., 2022).

Further, amenity availability driven by vertical forces and rather than community demands becomes problematic for health, since the availability of amenities in an environment can make decisions to undertake a behaviour become automatic rather than conscious. (Marek et al., 2021). Not only do these factors influence health individually, but studies have shown that they also tend to be spatially clustered together, potentially compounding each other’s effects through the creation of ‘addictive environments’ (Macdonald et al., 2018; Marek et al., 2021).

While much attention has been given to the spatial distribution of individual amenities, changes over time to inequalities in the retail landscape has not been studied extensively. Research conducted before 2017 showed that there are persisting socio-economic inequalities over time in the availability of takeaways and alcohol only outlets (Angus et al., 2017; Maguire et al., 2015). As well as inequalities in availability, negative temporal changes (such as losses of amenities and opportunities) in the built environment can affect mental health, evoking feelings of grief and disorientation in residents which reduce wellbeing, particularly in places which are already ‘left behind’ in an economic and political sense (Tomaney et al., 2024). Alongside this, the resulting stigma of living in a ‘declining’ neighbourhood can compound health effects through residents feeling anxiety, depression and psychosocial stress that their area is worse than it was (Smith and Anderson, 2018; Wutich et al., 2014), adding to the physical effects of being close to such amenities. Nonetheless, there have been very few, if any, studies which explore historical trends in availability of amenities in the most recent decade.

## 3 Aim

This paper explores the changing distribution of health-related amenities and the association with area level deprivation and region in England between 2014 and 2024. We include a range of health-related amenities, some extensively studied (takeaways, gambling out-lets, supermarkets, alcohol only outlets) and some that have not been researched previously (vape shops, pawnbrokers, public toilets).

## 4 Data and methodology

### 4.1 Study design

We used geographic data analysis and statistical modelling to describe the association between health-related amenities, area level deprivation and region across all of England between 2014 and 2024. We have three main outcomes, the availability of each amenity per 10,000 people for the descriptive analysis; the gain of a potentially health harming amenity; and loss of a health conducive amenity, which are used in the statistical models.

Our exposures are quintiles of area level deprivation by Middle Super Output Area (MSOA), which represents the hyperlocal “neighbourhood” contextual driver of amenity availability, and geographic region (North/South/Midlands), which capture some aspects of the wider socio-economic and political drivers of amenities. We also map amenities using Local Authority Districts as an ‘intermediate’ administrative scale between neighbourhood and region. As well as the spatial scale, we also consider the temporal scale to take into account the dynamic nature of places and amenity availability.

The covariates include the contextual classification of a place-urban/ rural and coastal/inland descriptors. We hypothesise that both deprivation quintile and amenity availability will vary depending on the geographical descriptor of a place. We also adjust the model based on the age and ethnicity structure of each place to account for any demographic or compositional drivers of availability that might be present.

### 4.2 Data

The locations of the amenities was obtained through the Ordnance Survey (OS) Points of Interest (PoI) datasets at yearly intervals from September 2014 to September 2024 (Ordnance Survey Points of Interest 2014-2024). The Ordnance Survey is the national government mapping agency of Great Britain and are the gold standard of geographic and cartographic data, developing and maintaining the definitive National Map of the UK (Ordnance Survey, 2025a). The Points of Interest Dataset is a comprehensive directory of all businesses, education and leisure services in Britain. It is sourced and cross-validated from more than 100 suppliers of listings (Ordnance Survey, 2025b). The data is available as points with coordinates, so it can be linked using GIS to other types of geographic data. This dataset is suitable for this analysis as we are confident it contains almost all registered amenities from a trusted mapping agency. While this data has been used extensively in geographic research, it has not been used to its full potential in a health research context (for examples, see: Hepburn et al., 2025; Hobbs et al., 2019). The amenities in this study were chosen primarily due to their link with health, as outlined in the literature review.

All analyses were undertaken at the Middle Super Output Area (MSOA) level, which is one of the statistical units used by the Office for National Statistics (ONS) for census data collection. An MSOA is the medium sized statistical unit and consists of 5000-15,000 residents or 2000–6000 households, often used for administrative and health data purposes in England and Wales. Since amenities often serve a larger area than the immediate residential population represented by a Lower Super Output Area (LSOA), MSOAs offer a better conceptual representation of a person’s local area and the amenities they can access. We used 6791 MSOAs covering all of England obtained from the ONS using the 2011 boundaries to enable us to link additional data.

We used the Index of Multiple Deprivation (IMD) 2019 which is the official measure of relative deprivation in small areas in England. It is based on 39 indicators which are combined into seven domains of deprivation: Income, Employment, Health and Disability, Education, Skills and Training, Crime, Barriers to Housing and Services and Living Environment. The 2019 release is the most recent one at the time of writing, and the IMD is not designed to measure absolute change in deprivation over time (Ministry of Housing, Communities & Local Government, 2019).

We also linked other geographical data on region on MSOA level (mySociety Research, 2019) and the Rural Urban Classification 2011 of MSOAs in England, which was developed by the Office for National Statistics (Office for National Statistics (ONS), 2011). The Rural-Urban Classification consists of two main categories, where an MSOA is urban if it is linked to a Built Up Area of over 10,000 residents, and rural otherwise. These categories are split further by building density and proximity to other built up areas. We use four categories in our analysis to reflect the diversity of neighbourhoods without being too granular: Urban conurbations, Cities and Towns, Towns and Fringe and Villages and Hamlets. Coastal status was obtained by linking MSOAs to the Coastal Built Up Areas classification developed by the ONS. (Office for National Statistics, 2021).

We used the Mid-year population estimates for 2014–2020 for MSOAs (Office for National Statistics (ONS), 2024) to calculate the provision per 10,000 people measure. We also included data from the 2011 census on age and ethnicity structure downloaded from the NOMIS database (Nomis, 2013). The median age of the population of the MSOA was used to represent the age structure, and the proportion of white people used to represent the ethnicity structure.

### 4.3 Geospatial analysis

We used Geographic Information System (GIS) analysis to generate the number of amenities per 10,000 population variables to compare availability across areas. To create the base map, the 2011 MSOA boundaries were linked using a spatial join to the IMD, urban/rural classification, region and MSOA population estimate in Quantum Geographic Information System (QGIS) Software (QGIS.org, 2025). Then, the OS dataset was filtered to the specific amenity required (as outlined in supplement A, Table S1) and the number of each amenity was represented as points within each MSOA polygon. The R package “sf” (Pebesma, 2018) was used to count the number of points within each polygon, and the tidyverse package (Wickham et al., 2019) was used to calculate the number of amenities per 10,000 people within the MSOA. We used a “for loop” to iterate the code over each yearly interval. Then, we summarised the mean amenity availability by deprivation and region and visualised it in heat maps on LAD level to compare availability.

### 4.4 Statistical analysis

We used statistical methods, in StataNow (StataCorp, 2025), to describe the change over time in the availability of amenities and test the associations between the availability of amenities and area level deprivation. Firstly, descriptive summary statistics of the availability of amenities were used to uncover time trends in the change between 2014 and 2024. Secondly, we used two logistic regression models to describe the association between the gain or loss of an amenity, region and deprivation. We tested the association between deprivation and gaining a potentially health harming amenity and deprivation and losing a health conducive amenity. This was determined using a change score of the difference between availability per 10,000 people in 2024 minus the availability in 2014. A positive value is a gain and is operationalised in the potentially health harming amenity models as a binary variable where a positive value equals 1. A negative value is a loss and operationalised in the health conducive amenity models as a binary variable where a negative value equals 1.

We adjusted for compositional and contextual factors in order to attenuate the association between deprivation and amenity gains and losses. The compositional factors that were available on MSOA level were age and ethnicity structure, which we adjusted for since such factors may influence the amenities available. Similarly, region, coastal status, and rural/urban classification all have an existing association with deprivation and can drive amenity availability, so we adjusted for them as well. Both deprivation and region were included to account for what has been referred to as “deprivation amplification”, where deprivation on different scales (i.e., individual, small area level, regional level deprivation) interacts and compounds, leading to area level deprivation having a larger effect in the Northern regions of England when compared to the rest of the country (Macintyre, 2007; Munford et al., 2022).

We also conducted a sensitivity analysis by only including MSOAs where there was at least one amenity, to exclude areas which had no amenities at all, to further account for factors which may influence amenity distribution.

## 5 Results

### 5.1 Trends in availability of amenities over 2014–2024 in England by IMD and region

As outlined in Table 1, there were significant inequalities by deprivation on the MSOA level in 2024 with higher availability of takeaways (15.8 per 10,000 vs 5.6 per 10,0000), off-licenses (1.0 per 10,000 vs 0.6 per 10,000), bookmakers (1.8 per 10,0000 vs 0.5 per 10,000), vape shops (0.7 per 10,000 vs 0.2 per 10,000) and pawnbrokers (0.5 per 10,000 vs 0.02 per 10,000) in the most deprived areas compared to the least deprived areas. All the potentially health harming amenities have higher availability in the most deprived areas.

**Fig. 1:**
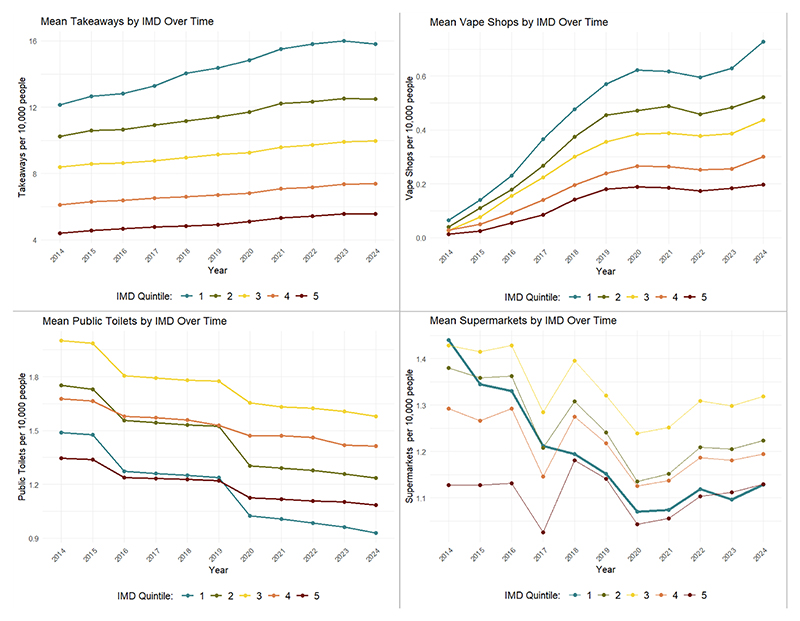
Change over time in selected amenities stratified by IMD quintile where IMD 1 is most deprived.

As seen in Fig. 1, and Supplementary Table S2, there are very clear inequalities in the availability of these amenities by deprivation. However, the change in availability over the last decade has been more variable. Takeaways (30 % vs 27 %) have increased at a higher rate in the most deprived areas when compared to the least deprived areas. The most deprived areas have seen a smaller decrease in some health harming amenities, such as bookmakers (–19 % vs –24 %) and pawn-brokers (–26 % v –43 %), when compared to the least deprived areas. Off licenses have decreased overall with a greater decrease in the most deprived areas (–27 % vs 15 %). Vape shops have seen over a tenfold increase in all areas, with a larger increase seen in the least deprived areas (1036 % vs 1576 %). Similarly, public toilets (–38 % vs –19 %) and supermarkets (–22 % vs 0 %) have been declining faster in the most deprived areas (see Fig. 1).

The maps in Fig. 3 show the patterning of three amenities on a Local Authority District scale. The highest concentrations of takeaways, pawnbrokers and bookmakers are found in the northern local authorities and in the more urban areas across the country. This is particularly pronounced in the ‘deindustrialised belt’ of cities in the North and Midlands such as Manchester, Leeds and Birmingham (noted as darkest blue on the maps).

**Fig. 2:**
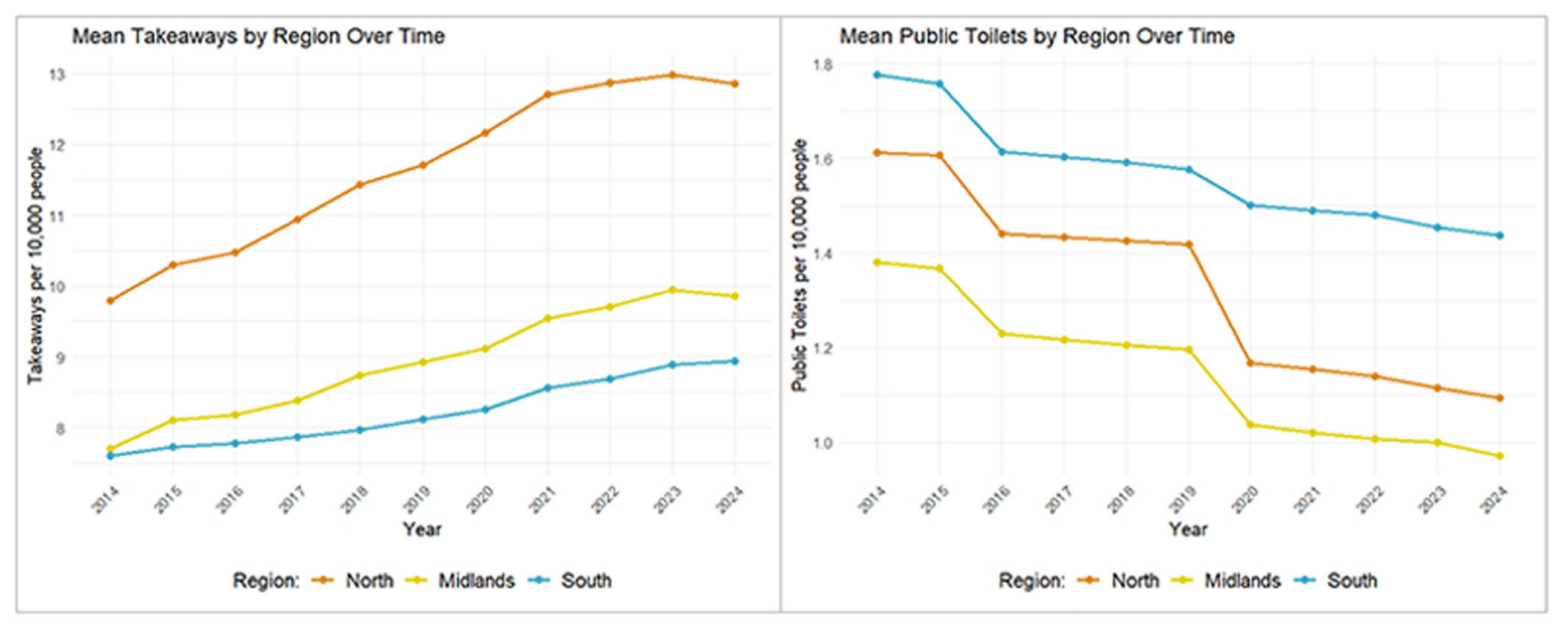
Change over time in selected amenities stratified by region.

**Fig. 3:**
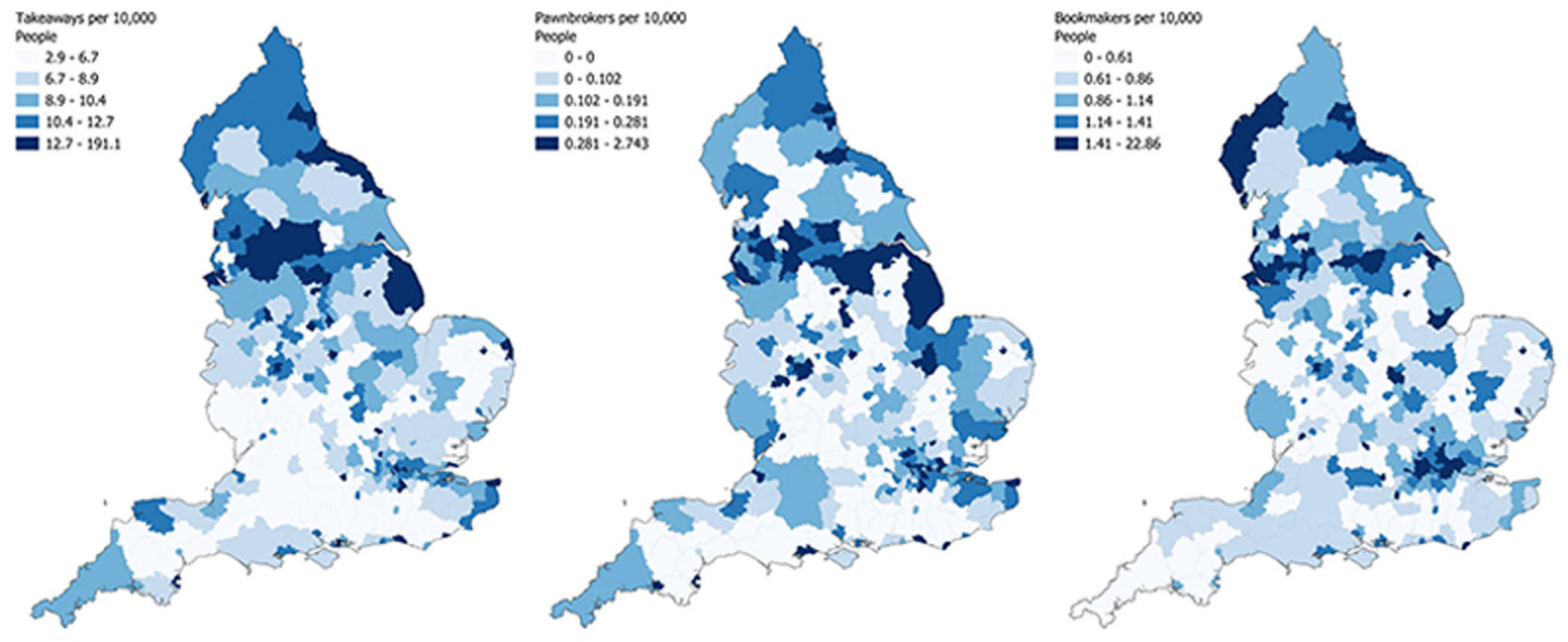
Maps on Local Authority District level of the availability of selected amenities in 2024.

The patterns that emerged on the hyperlocal and administrative scales was noted for regions in Table 2, where there is a higher availability in 2024 of takeaways (12.9 per 10,000 vs 9.0 per 10,000), off licenses (0.9 per 10,000 vs 0.8 per 10,000), bookmakers (1.3 per 10,0000 vs 1.0 per 10,000), vape shops (0.6 per 10,000 vs 0.3 per 10,000) and pawnbrokers (0.3 per 10,000 vs 0.1 per 10,000) in the North than there is in the South. The North has also seen a higher increase in takeaways (31 % vs 18 %) and a larger decrease in public toilets (–32 % vs –19 %) when compared to the South (Fig. 2).

As well as having higher availability in areas that are already disadvantaged, health-related amenities are highly clustered together within MSOAs as shown in Table 3. In neighbourhoods where there is a high availability of takeaways, there is also high availability of bookmakers (0.71), pawnbrokers (0.56) and vape shops (0.58). This pattern is similar for other potentially health harming amenities.

### 5.2 The association between amenity change over time, deprivation and region

The inequalities by deprivation in availability also extended to the change over time in health-related amenities, as outlined in Tables 4 and 5 and stepwise adjustment in Supplementary Tables S3–S9. The most deprived areas were more likely to gain a takeaway (OR 1.56 [1.28,1.91]), a bookmaker (OR 2.14[1.64,2.79]), a pawnbroker (OR 5.36 [2.56,11.22]) or a vape shop (OR 2.11[1.68, 2.66]) when compared to the least deprived areas. They were also more likely to lose a supermarket (OR 1.95[1.62,2.35]) or a public toilet (OR 1.34 [1.12,1.62]) when compared to the least deprived areas. Overall, the most deprived areas are more likely to gain potentially health harming amenities, and also more likely to lose health conducive ones.

These patterns were also present on a regional level as outlined in Tables 4 and 5 The North was more likely to gain a takeaway (OR 1.65 [1.44,1.90]), a bookmaker (OR 1.40 [1.18,1.66]), a pawnbroker (OR 1.72 [1.23,2.40]) or a vape shop (OR 1.30 [1.11,1.51]) when compared to the South. However, there was no association between region and losing a supermarket or public toilet.

Results from the stepwise addition of additional variables/confounders shows that the main effects remain qualitatively unchanged when other contextual and compositional factors are included (Supplementary Tables S3–S9).

The results of the sensitivity analysis in Supplementary Tables S10 and S11 show that across England, 97.6 % of MSOAs had at least one amenity of interest. The point estimates of the sensitivity analysis were within the original confidence intervals which indicates that the original estimates are robust to factors which mean there are no amenities at all in an MSOA.

## 6 Discussion

Our work presents one of the first spatio-temporal surveys of health-related amenities on the national level using the most up to date data between 2014 and 2024. Our study has found that on the neighbourhood scale, the most deprived areas were more likely to gain potentially health harming amenities, and also more likely to lose health conducive ones in the past ten years, when compared to the least deprived areas.

We also found that in 2024, availability of potentially health harming amenities (takeaways, off licenses, bookmakers, vape shops and pawnbrokers) was uniformly higher in the most deprived areas and the provision of health conducive amenities (supermarkets and public toilets) was uniformly lower. These findings are consistent with the literature on takeaways (Lamichhane et al., 2013; Maguire et al., 2015), off-licenses (Connor et al., 2011; Shortt et al., 2015), bookmakers (Pearce et al., 2008; Wardle et al., 2014) and supermarkets (Lamichhane et al., 2013; Maguire et al., 2015). Our findings on vape shops, pawn-brokers and public toilets contribute novel insights to the literature around health-related amenities.

The higher availability of potentially health harming amenities in more deprived areas and regions follows similar patterning of other health and socio-spatial inequalities. In fact, evidence suggests that some gambling and takeaway chains purposefully target deprived areas, where residents are already vulnerable to problem gambling and fast food consumption (Adeniyi et al., 2023; Cummins et al., 2005). Our findings also show that potentially health harming amenities are highly clustered together on a neighbourhood level - the areas which contain high availability of each also contain high availability of the other. This spatial clustering pattern is consistent with the literature and can amplify environmental risk factors in already socio-economically deprived areas. The clustering of such amenities also represents increased risk for negative health behaviours that may co-occur, such as problem drinking and gambling, which in turn creates addictive environments and cumulative health damaging exposures, further perpetuating environmental injustices (Macdonald et al., 2018; Schneider and Gruber, 2013). As well as changing the neighbourhood level context of places, the presence of clustering of such amenities also could indicate the local impact of political and economic processes at a larger regional scale (Cummins et al., 2007).

At the larger spatial scales, we do indeed find that spatial patterns found on the neighbourhood scale are mirrored on the local authority and regional level. The local authority maps showed that the areas which have highest concentrations of takeaways, bookmakers and pawnbrokers are in the North and primarily cities which experienced deindustrialisation and subsequent wealth inequalities (Pike et al., 2023; Tomlinson, 2021). On a regional level, similar patterns were observed with higher availability of takeaways, off licenses, bookmakers, vape shops and pawnbrokers in the North than there was in the South. We also found that the North was more likely to gain a takeaway, a bookmaker, a pawnbroker or a vape shop. The presence of these patterns on a regional scale indicates that decisions made on shop placement are not local and largely outside the influence of individuals and residents. Rather, that the wider economic and political context could be exerting a stronger influence than the compositional factors of a place in relation to amenity availability.

One of the vertical drivers of amenity availability is the financialisation of high streets and the proliferation of national and transnational retail chains, which moves away from the historic ‘community marketplace’ model of high streets which had previously served the needs of communities. This could make high streets and town centres more reliant on chain retailers. The collapse of a chain retailer, however, means that if one retailer collapses, all of their branches will close, affecting more areas than if an independent business closes. This is a manifestation of the loss and replacement of social infrastructures (including retail) in ‘left behind places’ (Tomaney et al., 2024) and leaves high streets sensitive to vertical drivers, including economic shocks.

Our results show that the spatio-temporal patterning of health-related amenity availability and change mirrors that of other geographic and health inequalities. While there has been national change in the quality of England’s high streets, some areas have fared worse than others. These are often the same areas which are considered ‘left behind’ and already suffer from high levels of health inequality, such as deprived areas, deindustrialised areas and the Northern regions. The same areas have also suffered from disproportionately large impact of austerity measures since 2010, where deindustrialised areas and less affluent coastal areas have had larger financial losses from welfare reform (Beatty and Fothergill, 2018). The causes, therefore, are the same longstanding economic and political inequalities in wealth and resource allocation - the “causes of the causes” of health inequalities (Bambra, 2016).

However, we must be mindful to avoid the unintentional stigmatisation of specific places by suggesting that their high streets have gotten worse or unhealthier, especially in this case where the provision of amenities is largely outside the control of residents. There is a large body of ‘worst high street/decline of the high street’ discourse seen in the national media; when specific places are mentioned it can shift the blame of such ‘decline’ onto the demographic composition of places while disregarding the vertical influences on amenity provision. Such stigmatisation can further affect people’s health, on top of the effects of their environment (Smith and Anderson, 2018; Wutich et al., 2014). In our study, we want to highlight that high street decline is a national issue but places that already suffer from inequalities have been the hardest hit.

Since amenity provision can be driven by economic and political inequalities in wealth allocation, there are political levers available to regulate amenities on a local and national level. Local planning authorities have powers to grant or deny permissions to establish retail outlets and can develop strategies to manage the proliferation of certain amenities. This was put into practice when Gateshead Council reduced the number of takeaways in specific areas affected by the policies (Brown et al., 2022), which showed that takeaway management zone policies can limit the impact of fast food consumption on population health (Rahilly et al., 2024). Further, national gambling legislation around Fixed Odds Betting Terminals and banning the use of credit cards led to reductions in bookmakers on high streets. However, while this reduction was visible nationally, there was still a high availability of bookmakers in the most deprived areas. While national legislation can play a role, there also needs to be local and regional action where possible.

Local high street rejuvenation projects can also help improve the quality and resilience of high streets. A recent report by the House of Lords Built Environment Committee (2024) suggests that the dominance of retail on high streets is a thing of the past, and that high streets should be easier and safer to access and contain more healthful amenities such as public toilets. They also recommend involving residents in decision-making around their high streets, which could tilt the balance of power dynamics to effect change in residents’ favour. While high street rejuvenation can make a difference to the quality of the built environment, care must be taken so the interventions are inclusive, equitable and appropriate for the residents. As highlighted by Garthwaite and Bambra (2018) – one participant in their study stated around the new high street rejuvenation work that “it’s nice but no good if you can’t afford a coffee’. These examples highlight the need for a place-based policy approach and corresponding powers and funding to managing the availability of health-related amenities on high streets.

Our study has several strengths. Firstly, we used data on a small area level of 6791 MSOAs across all of England, which enabled national coverage rather than focusing on one or two cities or case studies. Using MSOAs was also a more accurate representation of a person’s local area than the smaller LSOAs, since amenities often serve a larger area than the immediate residential population. Furthermore, we used the most up to date amenity data over the past decade, which enabled us to study the change over time of amenity availability. Our multi-scalar approach (MSOA, LAD, region and time) enabled us to link the hyperlocal ‘neighbourhood’ scale with regional economic and political processes that vary through space and time.

However, we also have a number of limitations. While we adjusted for the main geographic and compositional indicators, we could not adjust for all spatial factors so there may be underlying spatial or economic processes which are not included. There is also likely to be missing data in the amenities dataset, which we dealt with through external validation where possible. Additionally, while the study is strengthened by using both descriptive statistics and regression models to examine the distribution of amenities and the change over time, this can occasionally lead to different results depending on the method used (for an example see vape shops in Table 4 vs S2). This difference is due to two potential reasons. First, some areas might have seen many of the same type of amenity open, while others only few (or even one), and some areas have a lower starting point of amenity availability, leading to a seemingly larger percentage increase. Secondly, the regression analysis also adjusts for region in the same model. Therefore, this can differentiate the regions vs. deprivation quintile effects, whereas the descriptive trends pool all areas in each quintile together, regardless of region.

It is also important to note that the availability of an amenity does not always translate to individual access. Access is a multifaceted concept which includes both spatial and aspatial aspects (Ribot and Peluso, 2003). People also often have complex spatial routines in their day-to-day lives (Kwan, 2022) so may be able to access amenities at different times and origins. In our analysis, we look at one aspect of spatial access which is the availability within a person’s neighbourhood. This provides important insights into the quality of the built environment around a person, since if the amenities are not available then it becomes harder to access them.

## 7 Conclusions

The availability of amenities is one of the environmental factors that can affect people’s health, through the interaction of people with health-related amenities and the subsequent ability to undertake beneficial health behaviours. Our findings indicated that the most deprived areas are gaining health harming amenities and losing health conducive ones when compared to the least deprived areas. The spatial patterning of health-related amenities on both the local authority and regional scale is similar to that of existing geographic health inequalities, which speaks to the influence of the same longstanding economic and political inequalities in wealth and resource allocation driving amenity availability. Areas which are deprived, left behind or otherwise disadvantaged already have a higher burden of ill-health, and changes in health-related amenities risks perpetuating health inequalities further. Since the drivers of amenity availability are primarily structural, upstream solutions and policy levers are required. Policymakers are encouraged to intervene in an inclusive and equitable manner to manage amenity availability for areas and populations most at risk.

## Supplementary Material

Supplementary data to this article can be found online at https://doi.org/10.1016/j.socscimed.2025.118863.

## Figures and Tables

**Table 1:** Mean availability per 10,000 people of amenities in 2024 stratified by IMD.

Provision per 10,000 people in 2024 of amenities by deprivation quintile (IMD)						
	England Average	IMD Q1 (most deprived)	IMD Q2	IMD Q3	IMD Q4	IMD Q5
Takeaways	10.2	15.8	12.5	9.9	7.4	5.6
Off-licenses	0.9	1.0	0.9	0.9	0.7	0.6
Bookmakers	1.1	1.8	1.5	1.0	0.7	0.5
Vape Shops	0.4	0.7	0.5	0.4	0.3	0.2
Pawnbrokers	0.2	0.5	0.2	0.1	0.05	0.02
Public Toilets	1.2	0.9	1.2	1.6	1.4	1.1
Supermarkets	1.2	1.1	1.2	1.3	1.2	1.1

**Table 2:** Mean availability per 10,000 people of selected amenities in 2024 stratified by region.

Provision per 10,000 people in 2024 of amenities by region				
	England Average	North	Midlands	South
Takeaways	10.2	12.9	9.9	9.0
Off-licenses	0.9	0.9	0.8	0.8
Bookmakers	1.1	1.3	0.9	1.0
Vape Shops	0.4	0.6	0.5	0.3
Pawnbrokers	0.2	0.3	0.1	0.1
Public Toilets	1.2	1.1	1.0	1.4
Supermarkets	1.2	1.2	1.3	1.2

**Table 3:** Correlation matrix of within MSOA clustering of amenities.

	Takeaways	Off-licenses	Bookmakers	Vape Shops	Pawnbrokers	Public Toilets	Supermarkets
Takeaways	1						
Off-licenses	0.44	1					
Bookmakers	0.71	0.36	1				
Vape Shops	0.58	0.26	0.49	1			
Pawnbrokers	0.56	0.25	0.54	0.44	1		
Public Toilets	0.32	0.13	0.27	0.24	0.15	1	
Supermarkets	0.19	0.11	0.09	0.12	0.09	0.17	1

**Table 4:** Results of the logistic regression for the association between gaining an amenity associated with negative health outcomes and deprivation.

Gain of an Amenity	Odds Ratio	[95 % CI]
**Takeaways**		
IMD Quintile:		
4	1.15	[0.98,1.35]
3	1.19	[1.01,1.40]
2	1.34	[1.12,1.59]
1(most deprived)	1.56	[1.28,1.91]
Region:		
Midlands	1.31	[1.14,1.52]
North	1.65	[1.44,1.90]
**Off Licenses**		
IMD Quintile:		
4	1.06	[0.87,1.29]
3	1.10	[0.90,1.34]
2	0.98	[0.80,1.20]
1(most deprived)	0.87	[0.69,1.09]
Region:		
Midlands	1.09	[0.92,1.29]
North	1.13	[0.97,1.33]
**Bookmakers**		
IMD Quintile:		
4	1.41	[1.11, 1.81]
3	1.84	[1.45,2.33]
2	2.28	[1.79,2.89]
1(most deprived)	2.14	[1.64,2.79]
Region:		
Midlands	0.97	[0.80,1.18]
North	1.40	[1.18,1.66]
**Vape Shops**		
IMD Quintile:		
4	1.59	[1.29,1.98]
3	2.15	[1.75,2.65]
2	2.05	[1.65,2.53]
1(most deprived)	2.11	[1.68,2.66]
Region:		
Midlands	1.27	[1.08,1.49]
North	1.30	[1.11,1.51]
**Pawnbrokers**		
IMD Quintile:		
4	2.46	[1.12,5.41]
3	4.33	[2.09,8.97]
2	4.67	[2.26,9.63]
1(most deprived)	5.36	[2.56,11.22]
Region:		
Midlands	1.06	[0.71,1.58]
North	1.72	[1.23,2.40]

**Table 5:** Results of the logistic regression for the association between losing an amenity associated with positive health outcomes and deprivation.

Loss of a healthy Amenity	Odds Ratio	[95 % CI]
**Supermarkets**		
IMD Quintile		
4	1.17	[1.00,1.37]
3	1.50	[1.28,1.76]
2	1.40	[1.18,1.65]
1(most deprived)	1.95	[1.62,2.35]
Region:		
Midlands	1.20	[1.05,1.38]
North	1.04	[0.91,1.19]
**Public Toilets**		
IMD Quintile		
4	1.11	[0.96,1.30]
3	1.42	[1.22,1.66]
2	1.34	[1.13,1.57]
1(most deprived)	1.34	[1.12,1.62]
Region:		
Midlands	0.87	[0.76,1.00]
North	0.89	[0.78,1.01]

## Data Availability

The authors do not have permission to share data.

## Data sharing statement

The data used in this study are licensed from Ordnance Survey and PointX and can only be shared to users with an Edina Digimap Educational License.
